# Supplementary material for: CAN OF SPINACH, a novel long non-coding RNA, affects iron deficiency responses in Arabidopsis thaliana
Source: Front Plant Sci. 2022 Oct 5;13:1005020. doi: 10.3389/fpls.2022.1005020 (PMC9581158; doi:10.3389/fpls.2022.1005020)
Supplement: Supplementary file 1 [file DataSheet_1.docx]

Supplementary Material

# Supplementary Figures and Tables


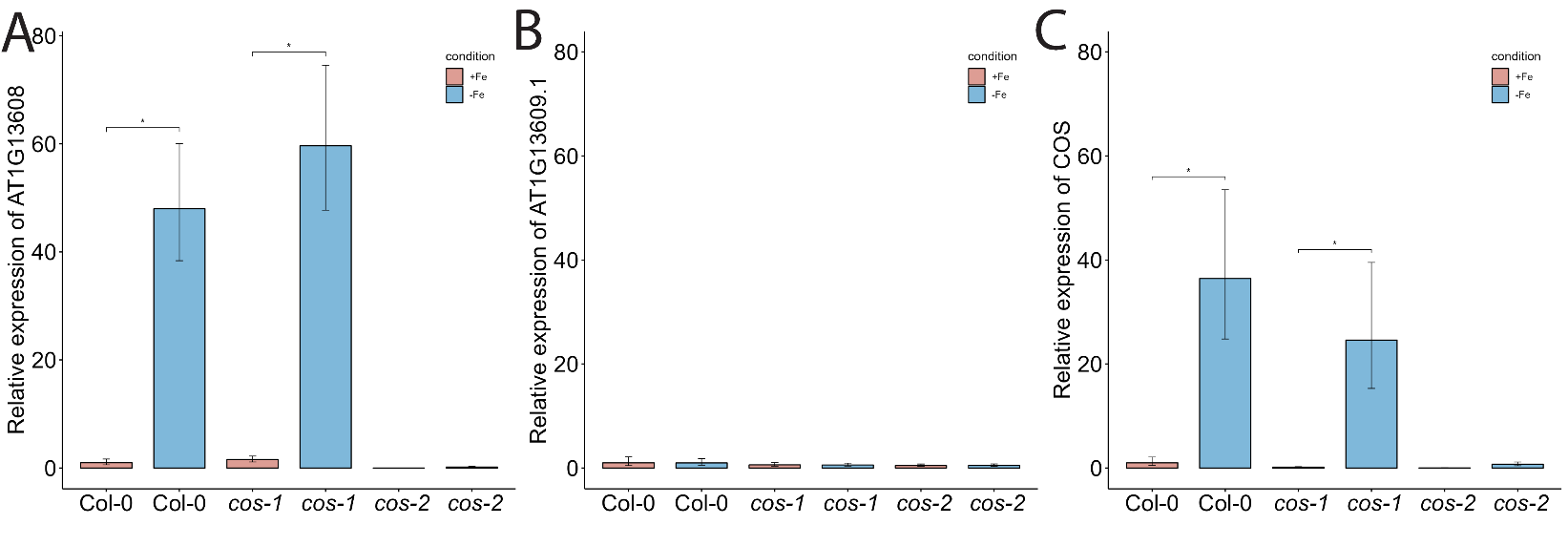


**Supplementary Figure 1.** Expression of genes in the *COS* locus in WT and *cos* mutants. Relative expression levels of AT1G13608 (A), AT1G13609 (B) and *COS* (C) in shoots of WT and cos mutants. Seedlings were grown on 1/2X MS agar with 50 μM Fe-EDTA for 14 d followed by a growth period of 3 d either on Fe-sufficient or Fe-deficient MS agar medium. Bars represent means ± SD of relative transcript levels normalized to *ACT2* (n=3).


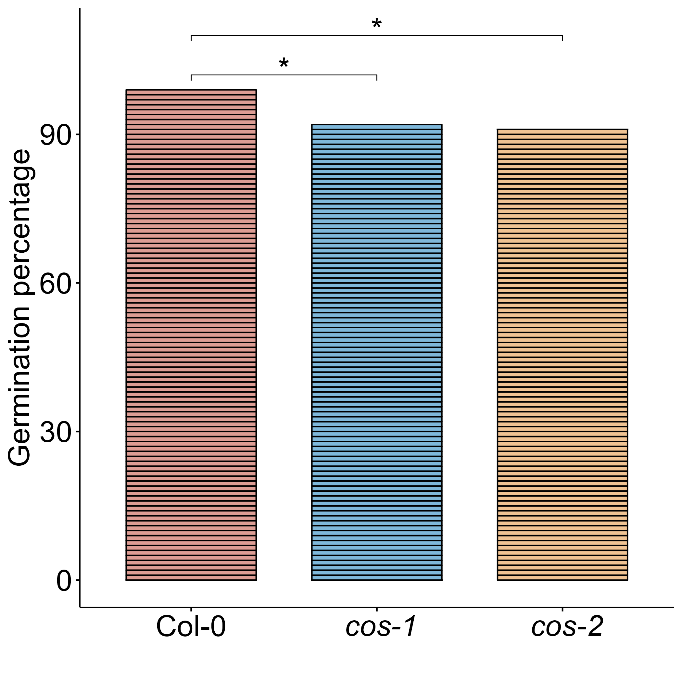


**Supplementary Figure 2.** Germination rate of WT and *cos* mutants. 100 seeds from each genotype were grown on 1/2X MS agar with 50 μM Fe-EDTA for four days and scored based on the emergence of hypocotyl.


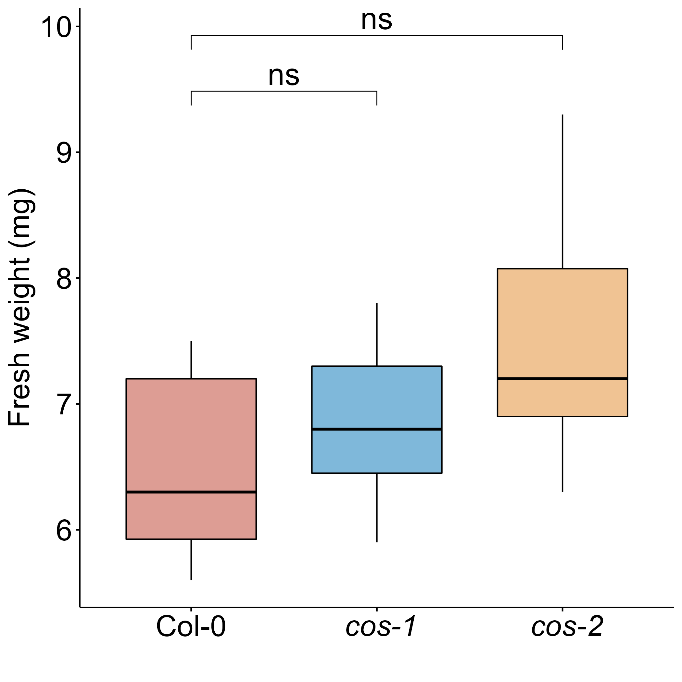


**Supplementary Figure 3.** Fresh weight of WT and *cos* mutants. Fresh weight of shoot systems of plants grown on soil for 14 d (n=6).

**
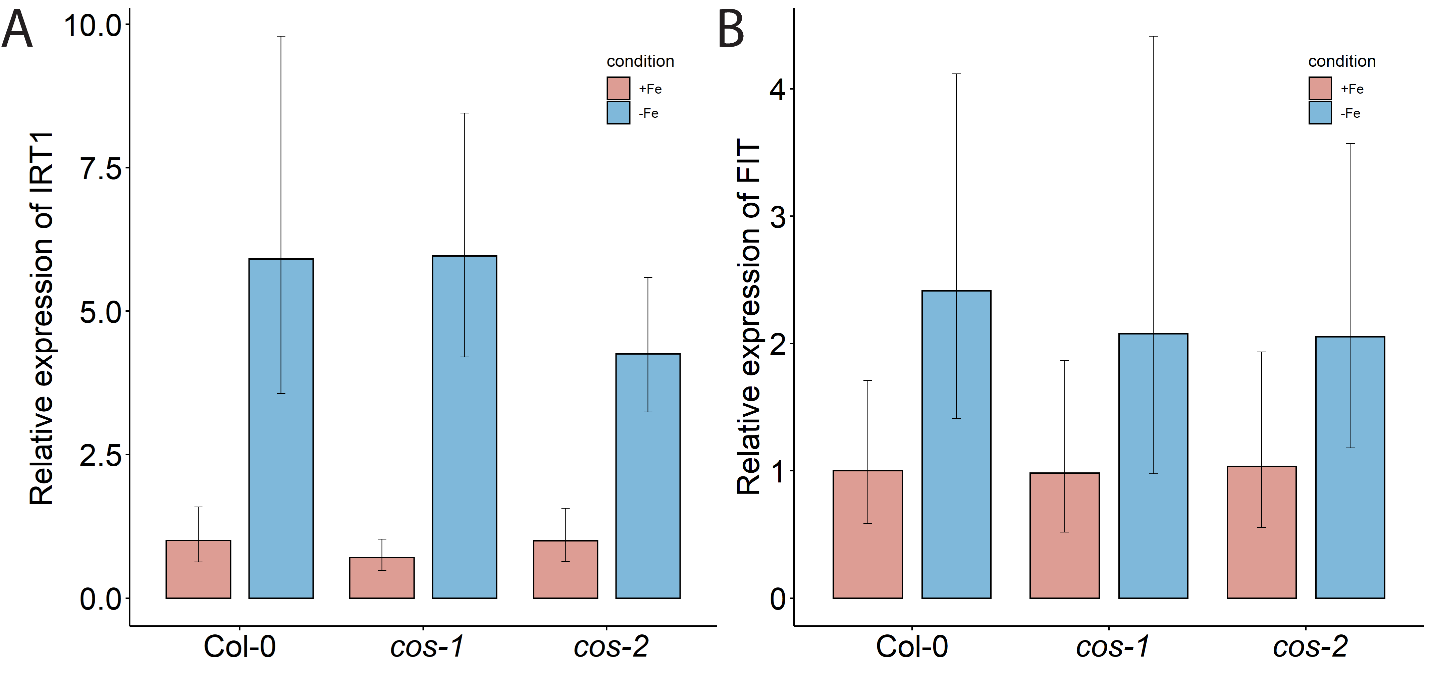
**

**Supplementary Figure 4.** Iron deficiency regulated gene expression in *cos* mutant roots. Relative expression levels of *IRT1* (A) and *FIT* (B) in roots of WT and *cos* mutants. Seedlings were grown on 1/2X MS agar with 50 μM Fe-EDTA for 14 d followed by a growth period of 3 d either on Fe-sufficient or Fe-deficient MS agar medium. Bars represent means ± SD of relative transcript levels normalized to *ACT2* (n=3).

**
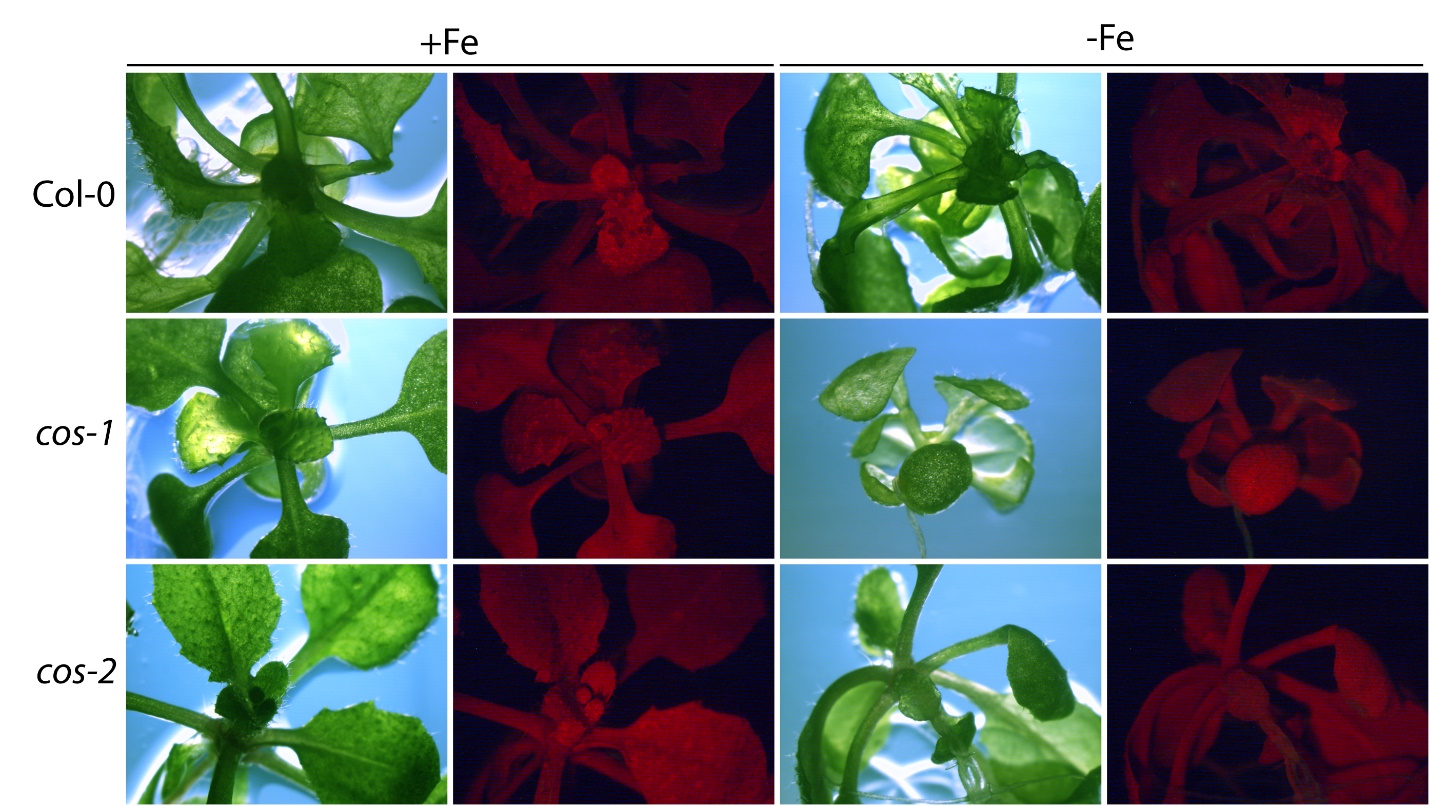
**

**Supplementary Figure 5.** Negative controls for SOSG treatment. Seedlings were grown on 1/2X MS agar with 50 μM Fe-EDTA for 14 d followed by a growth period of 3 d either on Fe-sufficient or Fe-deficient MS agar medium. WT and *cos* mutants were infiltrated with 50 mM phosphate buffer (pH 7.0), incubated under high light for 30 minutes and analyzed simultaneously with negative controls.

**Supplementary Table 1.** Sequences of primers used in this study

| Name | Sequence (5’ → 3’) | Reference |
| --- | --- | --- |
| COS-R1 | GGCTTCTTCTCATCCAGTTTACAAGC | This study |
| COS-F1 | GGCAACGAAACTTTCTGGACC | This study |
| COS-F2 | ACTCTCTACTTAACCATCATGCCCC | This study |
| COS-R3 | CGGTTGGATTGTTGGTTTCTGCT | This study |
| qRT-COS-F (5' end) | TGGCTTCTTCTCATCCAGTTT | This study |
| qRT-COS-R (5' end) | ACGTCATCTTTTCGTCTTTGCA | This study |
| qRT-COS-F (3’ end) | AGGTGTCCTAAGAATCTATGGCT | This study |
| qRT-COS-R (3’ end) | TCATTAACCACGTAACCGACACT | This study |
| qRT8-F (AT1G13608) | TCTCATCTCTGCTTGATCCTTT | This study |
| qRT8-R (AT1G13608) | GGATTTAATCACTAGTGGCAGTTT | This study |
| qRT9-F (AT1G13609.1) | TGTCTTCACAGCGACTGTTTCAG | This study |
| qRT9-R (AT1G13609.1) | GTGAGATTGTGACCTCACCCTTCC | This study |
| ACT2-forward | GCTGAGAGATTCAGATGCCCA | (Kumar et al., 2017) |
| ACT2-reverse | GTGGATTCCAGCAGCTTCCAT | (Kumar et al., 2017) |
| IRT1-forward | ACTTCAAACTGCGCCGGAAGAATG | (Kumar et al., 2017) |
| IRT1-reverse | AGCTTTGTTGACGCACGGTTC | (Kumar et al., 2017) |
| FER1-forward | CAACGTTGCTATGAAGGGACTAGC | (Kumar et al., 2017) |
| FER1-reverse | ACTCTTCCTCCTCTTTGGTTCTGG | (Kumar et al., 2017) |
| FIT-forward | TTTTCGCGGTATCAATCCTC | (Oh et al., 2016) |
| FIT-reverse | GGTATGTGTCCGGAGAAGGA | (Oh et al., 2016) |
| OXI1-forward | ACGACGCTAAATTGCTTGCT | (Akmakjian et al., 2021) |
| OXI1-reverse | CCGTGAAGAGACGGAAAGAG | (Akmakjian et al., 2021) |
| AT3G03810-forward | GCTTTGACAAGAGCCACCAA | (Akmakjian et al., 2021) |
| AT3G03810-reverse | CTACTGCACGTTTGCCAGACTT | (Akmakjian et al., 2021) |
| PYE-forward | CAGGACTTCCCATTTTCCAA | (Tissot et al., 2019) |
| PYE-reverse | CTTGTGTCTGGGGATCAGGT | (Tissot et al., 2019) |
| ILR3-forward | GCAACCTATTGGTGTTTCTTCTAACTC | (Tissot et al., 2019) |
| ILR3-reverse | CCAGGTTCTTTGCTAGCTTCTGA | (Tissot et al., 2019) |
| bHLH38-forward | AGCAGCAACCAAAGGCG | (Wang et al., 2007) |
| bHLH38-reverse | CCACTTGAAGATGCAAAGTGTAG | (Wang et al., 2007) |
| bHLH101-forward | CAGCTGAGAAACAAAGCAATG | (Akmakjian et al., 2021) |
| bHLH101-reverse | CAGTCTCACTTTGCAATCTCC | (Akmakjian et al., 2021) |
| IMA1-forward | ATGTCTTTTGTCGCAAACTT | (Mankotia et al., 2022) |
| IMA1-reverse | CACCACCATTCTCACTATATG | (Mankotia et al., 2022) |
| BTSL1-forward | GGCAATGAAGATGGATTTGG | (Mankotia et al., 2022) |
| BTSL1-reverse | TCATATGGAACCGTTGCTGA | (Mankotia et al., 2022) |

**Supplementary Table 2.** List of GO terms for Fe upregulated genes that have rapid response to Fe resupply

| GO biological process complete | # | # | expected | Fold Enrichment | P value |
| --- | --- | --- | --- | --- | --- |
| cellular response to iron ion starvation | 8 | 5 | .03 | > 100 | 3.09E-06 |
| regulation of iron ion transport | 7 | 3 | .03 | > 100 | 2.05E-02 |
| response to iron ion starvation | 16 | 4 | .06 | 64.09 | 3.04E-03 |
| cellular response to hypoxia | 239 | 23 | .93 | 24.67 | 2.58E-21 |
| cellular response to ethylene stimulus | 96 | 6 | .37 | 16.02 | 9.09E-03 |
| systemic acquired resistance | 116 | 6 | .45 | 13.26 | 2.56E-02 |
| response to molecule of bacterial origin | 117 | 6 | .46 | 13.15 | 2.69E-02 |
| response to chitin | 308 | 15 | 1.20 | 12.48 | 6.91E-09 |
| indole-containing compound metabolic process | 182 | 8 | .71 | 11.27 | 2.47E-03 |
| response to salicylic acid | 440 | 14 | 1.72 | 8.16 | 8.28E-06 |
| defense response to fungus | 702 | 19 | 2.74 | 6.94 | 1.34E-07 |
| response to wounding | 815 | 22 | 3.18 | 6.92 | 3.30E-09 |
| regulation of defense response | 744 | 19 | 2.90 | 6.55 | 3.48E-07 |
| response to oxidative stress | 613 | 15 | 2.39 | 6.27 | 6.62E-05 |
| defense response to bacterium | 1029 | 21 | 4.01 | 5.23 | 1.78E-06 |
| hormone-mediated signaling pathway | 930 | 15 | 3.63 | 4.13 | 1.17E-02 |
| response to water deprivation | 1000 | 16 | 3.90 | 4.10 | 5.97E-03 |
| regulation of DNA-templated transcription | 2184 | 23 | 8.52 | 2.70 | 3.28E-02 |

**Supplementary Table 3.** List of GO terms for Fe upregulated genes that don’t have rapid response to Fe resupply

| GO biological process complete | # | # | expected | Fold Enrichment | P value |
| --- | --- | --- | --- | --- | --- |
| removal of superoxide radicals | 11 | 3 | .02 | > 100 | 2.69E-03 |
| cellular transition metal ion homeostasis | 65 | 4 | .09 | 44.42 | 7.72E-03 |

**Supplementary Table 4.** List of GO terms for Fe downregulated genes that have rapid response to Fe resupply

| GO biological process complete | # | # | [expected](http://go.pantherdb.org/tools/compareToRefList.jsp?sortOrder=2&sortList=upload_1&sortField=exp) | Fold Enrichment | P value |
| --- | --- | --- | --- | --- | --- |
| response to oxidative stress | 613 | 9 | 1.36 | 6.60 | 2.73E-02 |
| response to abiotic stimulus | 4114 | 23 | 9.15 | 2.51 | 3.79E-02 |

**Supplementary References**

Akmakjian, G. Z., Riaz, N., and Guerinot, M. L. (2021). Photoprotection during iron deficiency is mediated by the bHLH transcription factors PYE and ILR3. *Proc. Natl. Acad. Sci. U. S. A.* 118, e2024918118. doi: 10.1073/pnas.2024918118.

Kumar, R. K., Chu, H. H., Abundis, C., Vasques, K., Rodriguez, D. C., Chia, J. C., et al. (2017). Iron-nicotianamine transporters are required for proper long distance iron signaling. *Plant Physiol.* 175, 1254–1268. doi: 10.1104/pp.17.00821.

Mankotia, S., Singh, D., Monika, K., Meena, H., Meena, V., Yadav, R. K., et al. (2022). Elongated Hypocotyl 5 (HY5) regulates BRUTUS (BTS) to maintain Iron homeostasis in Arabidopsis thaliana. *bioRxiv*, 2022.04.26.489524. doi: 10.1101/2022.04.26.489524.

Oh, Y. J., Kim, H., Seo, S. H., Hwang, B. G., Chang, Y. S., Lee, J., et al. (2016). Cytochrome b5 Reductase 1 Triggers Serial Reactions that Lead to Iron Uptake in Plants. *Mol. Plant* 9, 501–513. doi: 10.1016/j.molp.2015.12.010.

Tissot, N., Robe, K., Gao, F., Grant-Grant, S., Boucherez, J., Bellegarde, F., et al. (2019). Transcriptional integration of the responses to iron availability in Arabidopsis by the bHLH factor ILR3. *New Phytol.* 223, 1433–1446. doi: https://doi.org/10.1111/nph.15753.

Wang, H. Y., Klatte, M., Jakoby, M., Bäumlein, H., Weisshaar, B., and Bauer, P. (2007). Iron deficiency-mediated stress regulation of four subgroup Ib BHLH genes in Arabidopsis thaliana. *Planta* 226, 897–908. doi: 10.1007/s00425-007-0535-x.
